# Obesity and craniofacial variables in subjects with obstructive sleep apnea syndrome: comparisons of cephalometric values

**DOI:** 10.1186/1746-160X-3-41

**Published:** 2007-12-22

**Authors:** Antonino M Cuccia, Giuseppina Campisi, Rosangela Cannavale, Giuseppe Colella

**Affiliations:** 1 Department of Dental Sciences "G. Messina," University of Palermo, Palermo, Italy; 2 Department of Head and Neck Pathology, II University of Naples, Naples, Italy

## Abstract

**Background:**

The aim of this paper was to determine the most common craniofacial changes in patients suffering Obstructive Sleep Apnea Syndrome (OSAS) with regards to the degree of obesity. Accordingly, cephalometric data reported in the literature was searched and analyzed.

**Methods:**

After a careful analysis of the literature from 1990 to 2006, 5 papers with similar procedural criteria were selected. Inclusion criteria were: recruitment of Caucasian patients with an apnea-hypopnea index (AHI) >10 as grouped in non-obese (Body Mass Index – [BMI] < 30) *vs*. obese (BMI ≥ 30).

**Results:**

A low position of the hyoid bone was present in both groups. In non-obese patients, an increased value of the ANB angle and a reduced dimension of the cranial base (S-N) were found to be the most common finding, whereas major skeletal divergence (ANS-PNS ^Go-Me) was evident among obese patients. No strict association was found between OSAS and length of the soft palate.

**Conclusion:**

In both non-obese and obese OSAS patients, skeletal changes were often evident; with special emphasis of intermaxillary divergence in obese patients. Unexpectedly, in obese OSAS patients, alterations of oropharyngeal soft tissue were not always present and did not prevail.

## Introduction

Obstructive Sleep Apnea Syndrome (OSAS) is an obstructive-type respiratory disorder of sleep, associated with excessive drowsiness during the day or with at least two of the following symptoms: sudden awakening with a sensation of suffocation, not sufficiently refreshing sleep, and tiredness during the day and problems in the cognitive sphere. Apnea can be defined as an interruption of breathing during sleep, with persistence of thoracic and/or abdominal movements associated with a decrease in oxygen tension and a consequent desaturation of oxygen of the arterial hemoglobin [1].

The term hypopnoea means a decrease of >50% in airflow, with a persistence of the thoracic and/or abdominal movements. Hypopnea may also be defined as a reduction of breathing width (but >50%) associated to a reduction of oxygen saturation (SaO_2_) >3% or to an awakening.

According to the international standards, each of those respiratory events must last not less than 10 seconds and not more than 3 minutes. The frequency of apnea and hypopnea per hour of sleep is called "index of apnoea/hypoapnoea" or AHI. An AHI<5 is considered normal [2].

OSAS affects 2–4% of middle-aged men and 1–2% of middle-aged women in Western populations, although the majority of affected individuals remain undiagnosed [3,4].

Mostly males are affected, especially those who are obese or with abnormalities of the upper airway tract [5].

Apnea in females tends to appear later in life (usually after the menopause). On average, the degree of obesity associated with OSAS is higher than in males [6,7].

Some endocrinopathies are prone to OSAS. Hypothyroidism, in association with obesity, can help the onset; a mixedematous inhibition of the soft tissues of the upper respiratory tract (in particular the tongue); muscular hypotonia and acromegaly can favor the onset in association with macroglossia and problems in ventilatory control [8].

Abnormalities of the facial skeleton and of the soft tissues, in association with the narrowing of the upper respiratory airway, often lead to the onset of obstructive apnea.

The most frequent changes are: retrognathia, micrognathia, long face, inferior positioning of the hyoid bone, reduced cranial base length and angle, large ANB angle, steep mandibular plane, elongated maxillary and mandibular teeth, narrowing of the upper airway, long and large soft palate, and large tongue [9-18].

In obese patients who have a distribution of the body fat mainly over the upper part of their body, the resistance of the upper airway during sleep tends to be very high.

The Body Mass Index (BMI) is the measure of the obesity level of a subject. BMI equals a person's weight in kilograms divided by the height in square meters (BMI = Kg/m^2^) [19]. BMI is a widely used mean to define overweight. Although there is agreement about the general range of BMI that constitutes a "healthy" weight, agreement on an exact range has not been established with the range varying with age and gender. Ideally, healthy weight would fall within a range of BMI levels at which morbidity and mortality rates are lowest, and 'overweight' would be the BMI at which adverse effects increase [20]. BMIs are classified according to the standard BMI cut-off points. Accordingly, grades 1, 2 and 3 refer to undernutrition in adults in a sequence of 18.5, 17, 16 kg/m^2^. Overweight, obesity and severe obesity are in a sequence of 25, 30 and 40 kg/m^2 ^[21].

In light of these observations, the aim of this study was to search and compare the cephalometric data and mucosal oropharyngeal findings from publications on non-obese *vs*. obese Caucasian patients suffering OSAS.

## Methods

A thorough review of the relevant literature linking obstructive sleep apnea with cephalometric analysis was performed. The literature search was carried out using PubMed, SCIRUS and the Cochrane Central Register of Controlled Trials (CENTRAL). The search terminology used was: "OSAS and cephalometric analysis," and "OSAS and Body Mass Index."

Among the studies found, papers were selected on the basis of the following criteria:

studies on Caucasian patients, use of apnea-hypopnea index (AHI) to assess the presence of OSAS, the use of cephalometric analysis, and BMI evaluation of patients.

Only original papers (randomized and non randomized clinical trials, cohort studies, case-control studies and case report) published between 1990 and 2006 were selected for the review process.

It was decided to include the studies where the patients had an AHI >10 and where BMI ≥ 30 was considered obese, and a BMI <30 as non-obese.

The results were analyzed by comparing obese patients *vs *non-obese ones, in order to assess the most important variables present in the selected studies. The variables were considered as strictly related to apnea only if they did not show statistically significant differences among the papers selected.

### Statistical Analysis

All cephalometric variables analyzed in each study were expressed as Mean ± SD, and compared using One-way analysis of variance (ANOVA). When a significant difference was found, individual means were compared using the Student-Newman-Keuls test. In each study, the comparison of antropometric measurements (age, AHI and BMI) between obese and non obese was made with Student t-test. Data were analysed using statistical software (Primer of Biostatistics for Windows, version 4.02, McGraw-Hill Companies, New York) [22]. The level of significance was set at P < 0.05.

## Results

Although the PubMed search identified 269 items, only 25 studies appeared eligible for selection. Among these publications, 21 did not completely meet the criteria of inclusion and were excluded; leaving a total of 4 studies considered [21,23-25] eligible for inclusion in the present review.

The SCIRUS search identified 162 items (89 web results and 73 journal results). Following a thorough examination of 5 full-text articles that appeared eligible for selection, 4 were found irrelevant leaving only one study for [26].

**Figure 1:**
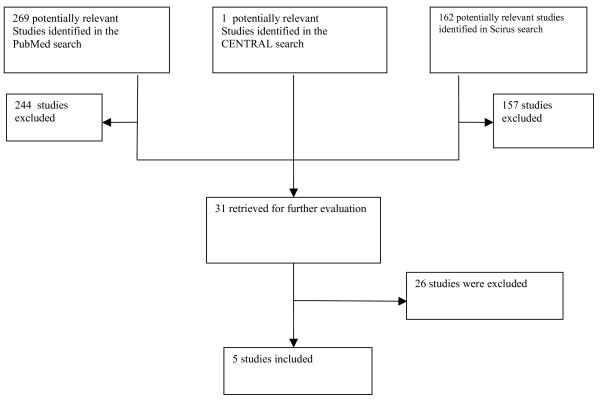
Flow diagram of the selection process of studies for systematic review on cephalometric analysis on nonobese OSA patients.

Cochrane Central Register of Controlled Trials (CENTRAL) provided one suitable result. Finally 5 studies were included in this review [21,23-26] (Fig. 1). Worthy of note, the only paper with a proper control group was published by Tangugsorn *et al.*[21]. The Sample size and anthropometric measurements of each study are shown in Table 1.

**Table 1:** Number of patients and antropometric measurements of each study

**Author**	Number of patients	**Non obese **(BMI <30) patients				**Obese **(BMI ≥ 30) patients									
		
		n	**Age (year)**	**BMI**	**AHI**	n	**Age (year)**	**BMI**	**AHI**	**Age**		**BMI**		**AHI**	
											
			Mean ± SD				Mean ± SD			t	P	t	P	t	P
**Pae et al. 1999**	17	9	54.44 ± 10.47	24.69 ± 1.86	48.45 ± 8.48	8	40.63 ± 12.61	39.34 ± 5.55	84.84 ± 31.44	*2.467*	*0.026*	*7.486*	*0.000*	*3.350*	*0.004*
**Paoli et al. 2000**	85	39	56 ± 11	26 ± 2	46 ± 23	46	54 ± 10	35 ± 5	50 ± 23	N.S.		*10.541*	*0.000*	N.S.	
**Tangugsorn et al. 2000**	100	43	48.3 ± 11.8	26.5 ± 2.7	32.2 ± 17.7	57	48.5 ± 11.7	34.3 ± 3.6	48.4 ± 28.8	N.S.		*11.900*	*0.000*	*3.252*	*0.002*
**Sforza et al 2000**	57	27	52.5 ± 9.8	-	62.1 ± 22.7	30	51.5 ± 8.3	-	82.2 ± 35.9	N.S.		-		*2.494*	*0.016*
**Iked et al 2001**	108	40	55 ± 11	24 ± 1.5	42 ± 24	68	54 ± 10	34.5 ± 4.7	46.6 ± 23.3	N.S.		*13.703*	*0.000*	N.S.	

### Cephalometric Measurements

When obese (BMI ≥ 30) individuals were compared to non-obese (BMI < 30) ones, mean age did not significantly differ in four studies. AHI differed significantly among three studies [21,23,24] and BMI showed significant differences among four studies [21,24-26] (Table 1). In particular, for non-obese patients, differences in mean age presents P = 0,019; differences for BMI presents P = 0,000; differences for AHI presents P = 0,000. For obese patients, all three characteristics presents P = 0,000 (Table 2).

**Table 2:** Comparison of age, BMI and AHI in in the selected studies.

Authors	Number of patients	Non-obese patients(BMI <30)				Obese patients (BMI ≥ 30)			
		
		n°	Age (year)	BMI	AHI	n°	Age (year)	BMI	AHI
					
			Mean ± SD				Mean ± SD		
**1 Pae et al., 1999**	17	9	54.44 ± 10.47	24.69 ± 1.86	48.45 ± 8.48	8	40.63 ± 12.61	39.34 ± 5.55	84.84 ± 31.44
**2 Paoli et al., 2000**	85	39	56 ± 11	26 ± 2	46 ± 23	46	54 ± 10	35 ± 5	50 ± 23
**3 Tangugsorn et al., 2000**	100	43	48.3 ± 11.8	26.5 ± 2.7	32.2 ± 17.7	57	48.5 ± 11.7	34.3 ± 3.6	48.4 ± 28.8
**4 Sforza et al., 2000**	57	27	52.5 ± 9.8	-	62.1 ± 22.7	30	51.5 ± 8.3	-	82.2 ± 35.9
**5 Iked et al., 2001**	108	40	55 ± 11	24 ± 1.5	42 ± 24	68	54 ± 10	34.5 ± 4.7	46.6 ± 23.3
***One-way ANOVA***		F	3.05	10.96	8.41	F	5.47	18.90	12.73
		P	0.019	0.000	0.000	P	0.000	0.000	0.000
		***SNK Post test***	**2 **vs **3**	**3 **vs **5**	**4 **vs **3**	***SNK Post test***	**2 **vs **1**	**1 **vs **3**	**1 **vs **5**
			**5 **vs **3**	**2 **vs **5**	**4 **vs **5**		**2 **vs **3**	**1 **vs **5**	**1 **vs **3**
					**4 **vs **2**		**5 **vs **1**	**1 **vs **2**	**1 **vs **2**
					**2 **vs **3**		**5 **vs **3**		**4 **vs **5**
					**5 **vs **3**		**4 **vs **1**		**4 **vs **3**
									**4 **vs **2**

Cephalometric values that showed statistical significance in obese patients were: ANB (P = 0,002), CVT^NSL (P = 0,000), S-Na mm (P = 0,000), H-Fh mm (P = 0,000), Length of soft palate mm (P = 0,000), Soft palate width mm (P = 0,024), Tongue width mm (P = 0,042), Inferior upper airway size mm (P = 0,047).

Cephalometric values that showed statistical significance in non-obese patients were: ANS PNS^GoMe (P = 0,017), H-Fh mm (P = 0,001), Length of soft palate (P = 0,000), Tongue length mm (P = 0,003), Tongue width mm (P = 0, 0016), Inferior upper airway size (P = 0,021).

With respect to the cephalometric measurements reported by all of the studies (i.e. SNA, SNB), no statistical differences were found between obese and non-obese individuals. A low position of the hyoid bone (H-GoMe) was present in both groups. In non-obese patients, an increased value of the ANB angle and a reduced dimension of the cranial base (S-N) was always evident. A major skeletal divergence (ANS-PNS ^Go-Me) was observed in the obese OSAS group. In summary, our data suggest that both in non-obese and obese OSAS patients, skeletal changes happen frequently and that in obese patients, soft tissue changes are not necessarily present and prevailing. In particular, obese OSAS patients present an increase in the intermaxillary divergence.

The other cephalometric parameters which could be compared totally or partially are shown in Tables 3 and 4.

**Table 3:** Comparison of cephalometric values in Obese Patients with OSAS

**PARAMETERS**	**1 **Pae (n 8)	**2 **Paoli (n 46)	**3 **Tangugsorn (n 57)	**4 **Sforza (n 30)	**5 **Iked (n 68)	***One-way ANOVA***			
						
						F	P	***SNK Post test***	
SNA°	81,75 ± 3,87	80 ± 4,4	80,59 ± 3,66	80,9 ± 4,5	80,1 ± 4,3	N.S.			
SNB°	77,00 ± 4,22	79 ± 4,3	78,24 ± 3,74	79,3 ± 4,6	79,2 ± 4,6	N.S.			
ANB°		0,4 ± 2,3	2,34 ± 2,83	1,6 ± 3,1	0,8 ± 3	4.96	0.002	**3 **vs **2** **3 **vs **5**	
SN^GoMe°	32,00 ± 5,26		33,18 ± 6,16	31,2 ± 6,0		N.S.			
Fh^GoMe°	26,75 ± 5,82	22 ± 6,6			21,7 ± 6,2	N.S.			
ANS PNS^GoMe°	21,81 ± 6,04	22 ± 7			22,4 ± 6,1	N.S.			
Goniac angle°		124 ± 5,3	123,16 ± 6,57		124,4 ± 4,6	N.S.			
NSBa°		131 ± 5,5	130,95 ± 4,89		130,8 ± 5,7	N.S.			
CVT^NSL °	155,63 ± 7,47		112,69 ± 6,94			363.92	0.000		
ANS-PNS mm			55,57 ± 3,45	54,1 ± 3,0		N.S.			
OVB mm	4,13 ± 2,40		3,40 ± 1,74			N.S.			
OVJ mm	4,81 ± 3,63		2,98 ± 2,75			N.S.			
S-Na mm		74 ± 3,6	71,4 ± 3,0	71,4 ± 3,0	72,9 ± 3,4	6.79	0.000	**2 **vs **3** **5 **vs **3**	**2 **vs **4** **5 **vs **4**
S-Ba mm		46 ± 3,8	46, 32 ± 3,45		45,8 ± 3,8	N.S.			
H-GoMe mm	25,56 ± 5,40	26 ± 6,7	27,48 ± 4,50	26,0 ± 5,9	25 ± 6	N.S.			
H-Fh mm		105 ± 6,9	107,94 ± 7,37		101,1 ± 7,9	13.20	0.000	**3 **vs **5** **2 **vs **5**	**3 **vs **2**
Length of soft palate mm		40 ± 4	52,01 ± 6,30	47,8 ± 5,0	39,1 ± 4,3	86.14	0.000	**3 **vs **5** **3 **vs **4** **4 **vs **2**	**3 **vs **2** **4 **vs **5**
Soft palate width mm			11,95 ± 1,84	12,9 ± 1,8		5.32	0.024		
Tongue length mm			86,37 ± 5,52	88,2 ± 5,3		N.S.			
Tongue width mm			41,25 ± 3,27	39,8 ± 2,8		4.25	0.042		
Superior upper airway size mm		7 ± 2,6		6,4 ± 2,5	6,7 ± 2,6	N.S.			
Inferior upper airway size mm		12 ± 4		11,5 ± 2,9	10,3 ± 3,8	3.12	0.047	**2 **vs **5**	
H-Ph mm		39 ± 5,4		38,7 ± 4,1	37,4 ± 5,7				
Lower facial height mm	76,25 ± 5,55			73,6 ± 5,0		N.S.			
Total facial height mm	132,13 ± 5,69			129,0 ± 6,4		N.S.			
GoMe mm		75 ± 5,2	74,29 ± 4,41		73,5 ± 4,9	N.S.			

**Table 4:** Comparison of cephalometric value in non-obese patients with OSAS

**PARAMETERS**	**1 **Pae (n 9)	**2 **Paoli (n 39)	**3 **Tangugsorn (n 43)	**4 **Sforza (n 27)	**5 **Iked (n 40)	***One-way ANOVA***			
SNA°	80,67 ± 4,12	79 ± 5,3	80,31 ± 4,83	82,2 ± 3,2	80,9 ± 3,5	F	P	***SNK Post test***	
SNB°	76,72 ± 3,29	77 ± 4,4	77,14 ± 4,90	78,7 ± 4,1	77,5 ± 3,8	N.S.			
ANB°		2 ± 2,8	3,16 ± 2,93	3,5 ± 2,8	3,3 ± 3,2	N.S.			
SN^GoMe°	28,33 ± 6,37		34,63 ± 9,53	32,7 ± 4,8		N.S.			
Fh^GoMe°	23,83 ± 6,90	24 ± 7,8			24,1 ± 6,3	N.S.			
ANS PNS^GoMe°	17,83 ± 4,73	25 ± 7,3			24,1 ± 6,3	4.31	0.017	**2 **vs **1**	**5 **vs **1**
Goniac angle°		124 ± 5,9	123 ± 6,57		121 ± 5	N.S.			
NSBa°		131 ± 5,5	130,95 ± 4,89		129,5 ± 5,1	N.S.			
CVT^NSL°	108 ± 9.50		107.23 ± 7.55			N.S.			
ANS-PNS mm			54,45 ± 3,62	53,9 ± 3,4		N.S.			
OVB mm	5,17 ± 1,62		4,04 ± 2,12			N.S.			
OVJ mm	5,28 ± 3,28		3,98 ± 2,33			N.S.			
S-Na mm		72 ± 3,5	71,09 ± 3,17	71,1 ± 3,0	71,9 ± 3,6	N.S.			
S-Ba mm		47 ± 2,6	45,87 ± 3,96		46,6 ± 3,1	N.S.			
H-GoMe mm	24,11 ± 9,98	24 ± 7	24,11 ± 5,71	25,4 ± 5,5	22,9 ± 5,5	N.S.			
H-Fh mm		104 ± 6,4	103,99 ± 7,46		99,2 ± 5,9	7	0.001	**2 **vs **5**	**3 **vs **5**
Length of soft palate mm		40 ± 4,8	47,46 ± 5,66	46,4 ± 4,7	38,8 ± 4,2	30.86	0.000	**3 **vs **5** **4 **vs **5**	**3 **vs **2** **4 **vs **2**
Soft palate width mm			11,61 ± 1,79	12,4 ± 2,0		N.S.			
Tongue length mm			82,30 ± 7,03	87,1 ± 5,2			0.003	-	
Tongue width mm			40,91 ± 3,85	38,0 ± 6,0		6.13	0.0016	-	
Superior upper airway size mm		6 ± 2,2		6,0 ± 2,2	5,6 ± 2	N.S.			
Inferior upper airway size mm		9,69 ± 2,5		10,8 ± 3,3	8,8 ± 2,8	4.03	0.021	**4 **vs **5** **2 **vs **5**	**4 **vs **2**
H-Ph mm		35 ± 3,8		35,0 ± 3,7	33,2 ± 4,2	N.S.			
Lower facial height mm	69,50 ± 5,70			73,4 ± 6,7		N.S.			
Total facial height mm	127,39 ± 8,16			128,8 ± 6,4		N.S.			
GoMe mm		75 ± 5,2	74,29 ± 4,41		73,2 ± 6,6	N.S.			

Iked *et al.*[26] published the data on 40 normal-weighted patients with apnea and 68 obese apnoeic patients, but did not compare them. The results of the comparison of the data are presented in Table 5.

**Table 5:** Comparison among subjects with AHI > 10 of obese and non-obese patients in Iked et al

Measurement	AHI<10, BMI<30 (n = 40)	AHI<10, BMI ≥ 30 (n = 68)	t	P
SNA°	80.9 ± 3.5	80.1 ± 4.3	NS	
SNB°	77.5 ± 3.8	79.2 ± 4.6	-1.974	0.05
ANB°	3.3 ± 3.2	0.8 ± 3	4.080	0.000
Tweed°	24.1 ± 6.3	21.7 ± 6.2	NS	
PMA°	24.2 ± 5.5	22.4 ± 6.1	NS	
NSH°	91.6 ± 4.7	91 ± 4.9	NS	
NSC°	114.1 ± 5.4	115.6 ± 6.1	NS	
AMH°	30.4 ± 7.6	31.1 ± 8.5	2.501	0.014
Na-S-Ba°	129.5 ± 5.1	130.8 ± 5.7	NS	
S-Na-Ba°	19.5 ± 2.6	18.6 ± 2.3	NS	
Na-Ba-S°	30.8 ± 3.1	30.5 ± 3.7	NS	
I/Fr°	106.3 ± 7.4	110.2 ± 9.2	-2.281	0.025
i/MP°	94.2 ± 7.3	92.7 ± 8.7	NS	
I/i°	135.2 ± 9.5	135	NS	
Goniac°	121.7 ± 5	124.4 ± 4.6	-2.852	0.005
S-Na mm	71.9 ± 3.6	72.9 ± 3.4	NS	
S-ba mm	46.6 ± 3.1	45.8 ± 3.8	NS	
VPS mm	5.6 ± 2	6.7 ± 2.6	-2.303	0.023
LPS mm	8.8 ± 2.8	10.3 ± 3.8	-2.172	0.032
HPS mm	33.2 ± 4.2	37.4 ± 5.7	-4.054	0.000
SPL mm	38.8 ± 4.2	39.1 ± 4.3	NS	
H-me mm	45.6 ± 6.3	49.6 ± 5.9	-3.318	0.001
PNS-A mm	49.4 ± 3.7	49.2 ± 3.5	-2.080	0.040
Go-Me mm	73.2 ± 6.6	73.5 ± 4.9	NS	
HPM mm	22.9 ± 5.5	25 ± 6	NS	
H-Fr mm	99.2 ± 5.9	101.1 ± 7.9	NS	
H-Bispinal mm	75.2 ± 5	77.2 ± 8	NS	
Na – H mm	58 ± 11	56.1 ± 9.6	NS	
H – BaNa mm	89.9 ± 7.2	93.1 ± 9	NS	
FLM %	44.1 ± 2.6	44 ± 2.4	NS	
FLI %	55.9 ± 2.6	55.9 ± 2.4	NS	

The number of patients and the anthropometric measurements of each study are shown in Table 1. When each study was analyzed with regard to the obese (BMI ≥ 30) and the non-obese (BMI < 30) individuals, significant differences were found for BMI (not available in Sforza), average age and AHI (Table 2). Unfortunately, only 3 cephalometric measurements (SNA, SNB e H-GoMe) were reported by all selected studies. From their comparisons, no significant differences were found between the obese and non-obese. The other comparable (or partially comparable) cephalometric parameters are shown in Table 3 and 4.

## Discussion

The present study compared the cephalometric variables of five publications [21,23-26], considering variables strictly related to OSAS. The variables particularly taken into account were: ANB, SNA, SNB, H-GoMe, ANSPNS ^GoMe, S-Na, length of the soft palate and CVT ^NSL. All selected publications were conducted on male patients, and they had the common aim of evaluating the cranio-cervical-facial skeletal characteristics and the soft tissues features in the upper airway of the cranium in OSAS patients with an AHI >10; in accordance with the BMI.

Sforza *et al.*[23]. have found that a long soft palate, an increased diametre of the neck and low position of the hyoid bone mainly affect the critical pharyngeal pressure- a measurement evaluating the degree of individual collapsibility of the upper airway.

All the selected publications individually reach the following common conclusions: non-obese OSAS patients have more risk to experience alterations in their bone structures, while obese individuals have more risk to confront changes in the soft tissues (i.e. length of the soft palate), while often retaining normal cranio-facial structures. In our analysis, we confirmed this datum as regards the skeletal class (ANB); and in particular we found that ANB does not play an important role in the genesis of OSAS in obese patients, while the same parameter appears to be important in the non-obese, as reported singulary by the authors cited above.

Furthermore, it was found a normal position of the upper jawbone in both groups and a slight retroposition of the mandible in non-obese patients *vs *obese OSAS patients, as investigated by SNA and SNB values. The hyoid bone is located in a lower position in OSAS patients (at the level of cervical vertebrae C4-C6) than in healthy subjects (C3-C4 level). Moreover, the hyoid bone in older OSAS subjects tends to be located in a lower position than in younger ones [26,27].

Although Tangugsorn *et al.*[21] found a significantly lower position of the hyoid bone in the obese patients, the position of the hyoid bone in obese and non-obese OSAS patients in all of the studies selected, was uniformly lower as confirmed herein by the lack of significance. According to Paoli *et al.*[25] the low position of the hyoid bone could be explained as an abnormality following OSAS more than a pre-existent or causative anatomical abnormality. Probably, over a long period of time, the repeated pressure at night-time causes a lengthening of the hyoid ligaments. Sforza *et al.*[23] consider that obesity, through the depositing of fat around the neck, could be the cause of further downward movement of the hyoid bone, hence altering the pharyngeal function and determining an easier collapsibility of the upper airway. Ferguson et al. reported that the distance between the hyoid bone and the mandibular plane increases in proportion to the circumference of the neck [28]. In agreement with Tangugsorn *et al.*[21], Nelson *et al.*[29], found the hyoid bone in a lower position in obese patients, considering this event as an adaptation to the increased size of the tongue. In our analysis, the intermaxillary divergence (ANSPNS ^GoMe) did not seem to play an important role in the development of OSAS in non-obese patients, while the same parameter appears to be important in obese patients.

The dimensions of the cranial base (S-Na) reveal an association with OSAS in non-obese subjects, which is in accordance with the literature [9,12], demonstrating a shorter dimension of the cranial base in such patients. On the contrary, such datum does not present association with obesity in the development of apnea.

Statistically significant differences emerged by analyzing the individual data of the soft palate of each study within obese and non-obese groups; discouraging the associability of such a value with apnea in both groups. The datum on head posture was reported only in the studies of Pae *et al.*[24] and Tangugsorn *et al.*[21]. The values reported in the latter two studies are higher than the normal values used as reference (97 ± 6) [30]. The comparison of the values CVT and NSL in obese patients shows a significantly higher value in the study of Pae *et al.*[24]. This result could be correlated to the higher values of BMI (39, 34 ± 5.55 vs 34.3 ± 3.6, P = 0.000) and AHI (84, 84 ± 31, 44 vs 48.4 ± 28.8, P = 0.000) in the sample of the latter study [24].

In this regard, several studies have shown that obstructions in the upper airway are connected with a variation in the head posture and with an increased cranio-cervical extension in order to increase the dimension of the airway [31,32]. Furthermore, Winnberg *et al*. [33] have shown that a hyper-extended head posture corresponds to a lower position of the hyoid bone.

The results of the present study show that obese OSAS patients have higher AHI values, even if the ages of obese patients are similar to those of non-obese individuals. In the study by Pae *et al.*[24], the obese patients were even younger [40, 63 ± 12, 61 vs 54, 44 ± 10.44, P = 0.026]). This confirms that obesity is more realistic as a risk factor than age for the development of OSAS [34,35], and the loss of weight one of the most valid therapies.

The limitations of our analisys include: the lack of information about the width of the soft palate, the tongue volume, thickness of the tissues around the pharynx and neck diameter which are all fundamental data to highlight the role of the soft tissues in the development of apneas.

In conclusion, the present study found that in non -obese as well obese OSAS patients, skeletal changes are often evident, especially in obese (in terms of intermaxillary divergence), and that, unexpectedly, in obese OSAS patients alterations of oropharyngeal soft tissue are not always present and prevailing.
